# Navigating the dichotomy: The top public servant's craft

**DOI:** 10.1111/padm.12600

**Published:** 2019-06-13

**Authors:** Erik‐Jan van Dorp, Paul ’t Hart

**Affiliations:** ^1^ Utrecht University School of Governance Utrecht University Utrecht The Netherlands

## Abstract

How in their day‐to‐day practices do top public servants straddle the politics–administration dichotomy (PAD), which tells them to serve and yet influence their ministers at the same time? To examine this, we discuss how three informal ‘rules of the game’ govern day‐to‐day political–administrative interactions in the Dutch core executive: mutual respect, discretionary space, and reciprocal loyalty. Drawing from 31 hours of elite‐interviews with one particular (authoritative) top public servant, who served multiple prime ministers, and supplementary interviews with his (former) ministers and co‐workers, we illustrate the top public servants’ craft of responsively and yet astutely straddling the ambiguous boundaries between ‘politics’ and ‘administration’. We argue that if PAD‐driven scholarship on elite administrative work is to remain relevant, it has to come to terms with the boundary‐blurring impacts of temporal interactions, the emergence of ‘hybrid’ ministerial advisers, and the ‘thickening’ of accountability regimes that affects both politicians and public servants.

## 1 INTRODUCTION

Much of what public administration researchers know about top public servants traditionally comes from institutional analysis (Raadschelders and Van der Meer 2014) and survey research (Hammerschmid et al. 2017) shedding light on constitutional and organizational settings, reward structures, as well as demographic features and attitudes. This, however, does not reveal much about what it is these elite public servants actually do—how they navigate the institutional contexts (values, norms, roles, rules, relationships) in which they operate. There is a much smaller, but important, trickle of research that focuses on the work practices of top officials, but many key questions about this work still loom large. What do they spend their time on and why (Fleming 2008; Van Dorp 2018)? How do they interpret their open‐ended job descriptions and enact their roles and responsibilities (Noordegraaf 2000)? How do they exercise influence while at the same time serving political office‐holders? How do they deal with professional and ethical dilemmas?

Answering such questions requires more intimate knowledge, for example, from in‐depth thematic or case study focused interviewing, diaries, and, ideally, observation. We need this type of research if we are to get any grip on the question how generic institutional norms such as the ‘politics–administration dichotomy’ or container notions such as ‘public service bargains’ (Hood and Lodge 2006) are understood, applied and negotiated by the people performing these roles on a day‐to‐day basis (Kaufman 1981; Rhodes 2011). However, because of prevailing norms of relative invisibility and illegibility, such data are hard to come by (also Boswell et al. 2018).

Our article nevertheless stands in this latter tradition. We ask how top public servants actually navigate the politics–administration dichotomy: how do they combine the ‘serving’ that it implies with the ‘influencing’ that is also part of the contemporary public servants’ job descriptions? In other words, we ask how ‘administrative responsiveness’ and ‘political astuteness’ take shape in day‐to‐day administrative work and behaviour. We investigate these questions in the context of the Dutch core executive. We use a qualitative approach that allows us to get an in‐depth understanding of senior public servants’ everyday lives and practices (informed by an ethnographic sensitivity, e.g., Rhodes et al. 2007; Watson 2011).

Based on our findings, we make two main contributions. First, we provide an all too rare up‐close and personal account of what working at the top of government feels like and to the people doing the job. Second, we apply a ‘craft’ perspective on public service work, moving beyond measuring public servants‘ attitudes and instead learning about their beliefs, practices and dilemmas by observing them at work in particular institutional settings (see also Rhodes 2016).

We begin by reviewing the theory and practice of political–administrative interaction We then describe our methods, followed by three case studies of one of the Netherlands’ most senior public servants being confronted with challenging situations that defy a ‘business as usual’ approach to navigating the politics–administration dichotomy. We conclude by drawing inferences about how responsiveness and astuteness are understood and performed by public service elites in times of turbulence.

## 2 POLITICS AND ADMINISTRATION IN CONTEMPORARY CORE EXECUTIVES

Practitioners and scholars alike have long noted and emphasized the distinctions—but also the points of overlap—between politics and administration (Waldo 1948; Rutgers 1997). Inspired by Wilson (1887), Goodnow (1900) and Weber (Gerth and Mills 2009), many have debated the ‘political–administration dichotomy’ (PAD). Wilson and Goodnow argued that the political realm needed a neutral and efficient administration as counterpart, whereas Weber was more concerned with securing effective political direction of powerful professional bureaucracies. Over the years, many have debated the ideal‐typical, moral, constitutional or empirical nature of the ‘dichotomy’. Empirical research across time, space and types of political systems keeps telling us that the hybrid models of political–administrative interaction are better generic descriptors of the ‘rules of the game’ governing the practice of political–administrative relations than the, essentially normative, model of the dichotomy (Aberbach et al. 1981; Peters 1988; Hartley et al. 2015; Alford et al. 2017). Svara (2001: 180) claims the ‘dichotomy’ is a ‘myth’, arguing that ‘complementarity’ of political and administrative work is in fact the salient norm, as expressed in ‘ongoing interaction, reciprocal influence, and mutual deference between elected officials and administrators’. Rhodes (2011) proposes the term ‘political‐administrators’ as a generic descriptor for ministers and top departmental officials alike, reflecting the shared set of beliefs and traditions of these elites operating at the heart of the core executive.

While students of core executive behaviour may be tempted to ‘ditch’ the politics–administration dichotomy altogether and instead assume, propagate and investigate political–administrative interdependence, complementarity and bargaining, something important, a constitutive element of public administration scholarship (Overeem 2012) and practice, would be lost. In many public service systems versions of the dichotomy continue to serve, explicitly or implicitly, as frames of reference for their institutional design as well as for the socialization and training of public servants into an ethic of neutral competence (Heclo 1975; Van der Meer et al. 2015). It forms the DNA of senior public servants, and shapes how they navigate ‘the political dimensions’ of their work.

### 2.1 Dutch political–administrative interaction: three rules of the game

It is in this spirit—mindful of the realities of hybridity, interdependence and bargaining but equally mindful of the continuing influence of the dichotomy in contemporary core executive practices—that we set out to examine political–administrative interactions at the apex of the Dutch public service. Dutch political–administrative relations evolve around three rules of the game that are partly based in constitutional law and partly grounded in evolving core executive practices: mutual respect, discretionary space and reciprocal loyalty (see Table 1) (Nieuwenkamp 2001; ’t Hart and Wille 2006; Hupe 2007).

**Table 1:** Rules of the game of political–administrative interaction in the Netherlands

Mutual respect	Elected office‐holders and public servants expect mutual respect. From public servants to the politics of the day, and from the elected office‐holders to the institutions they represent.
Discretionary space	Both politicians and public servants need to be able to legitimately exercise forms of public leadership in accordance with their respective roles and in the interest of the public.
Reciprocal loyalty	Public servants are loyal to the office of the minister. This loyalty rests on the precepts of ministerial responsibility and the reciprocal loyalty towards the public service.

Adapted from ‘t Hart and Wille (2006).

Mutual respect between elected office‐holders and public servants for the roles that each have to play within the larger constitutional framework of government is a bedrock norm. Although formally, Dutch doctrine is about the loyalty of public servants vis‐à‐vis ministers, empirical research has found that, in practice, public servants also expect a degree of in‐role loyalty of ministers vis‐à‐vis themselves and the value of the institution that they in turn represent.

Discretionary space refers to the idea that both politicians and administrators need to be able to legitimately exercise public leadership in accordance with their respective roles. Ministers are to do so at front stage, in what is understood to be the political realm: cabinet, parliament, the media, making substantive as well as tactical political calls on general policy directions as well as specific dossiers. Politicians have considerable discretion—limited only by the law, parliamentary support and, increasingly, media coverage—to do so. In Dutch constitutional theory, public servants have little to no such discretion. And yet in practice a norm has evolved that stipulates that ministers should refrain from micro‐managing their departments, allowing top‐level public servants to lead the ‘administrative’ processes of policy design, advice‐giving, policy implementation, evaluation and learning. NPM‐era reforms such as agencification have further cemented this norm (notwithstanding the fact that various agencies have been brought back firmly into the real of ministerial oversight over the years; Van Thiel and Yesilkagit 2014).

Reciprocal loyalty is rooted in the idea that the public service is expected to be loyal first and foremost to the office of minister and the constitutional norm of ministerial responsibility, not to the person of the office‐holder per se. In other words, only to the extent that ministers comport themselves responsibly in enacting their roles in cabinet, parliament, the media and at the ministry are they entitled to expect public servants to serve them to the hilt. But when ministers adopt a pattern of behaviour that public servants interpret as either violating the precepts of ministerial responsibility—for example, lying to parliament—or as violating the expectation of reciprocal ministerial loyalty towards the public service—for example, buck‐passing blame to them when publicly accounting for failed policies (Hood 2010; De Ruijter 2019)—the gloves can come off.

### 2.2 ‘Managing up’: balancing responsiveness and astuteness

We focus on one element that epitomizes the challenges top public servants face in navigating the interface between the professional and the political: ‘managing up’ to their political masters (Hartley et al. 2015; Van Dorp 2018). ‘Managing up’ can be distinguished from managing ‘down’ (leading an organization, building organizational capacity) and managing ‘out’ (engaging in network (meta‐)governance, communities, and in the public eye).

Elite administrators are supposed to be loyal to the minister but also deliver ‘frank and fearless’ advice. They have to perennially assess what works and what is appropriate (March and Olsen 1989; Rhodes 2016). The key challenge is to simultaneously be responsive to the government of the day (Wilson 2016, pp. 66–71) and serving its office‐holders (e.g., catering to their needs, giving advice upon request, providing order and structure to their hectic working days) while being politically astute (Hartley et al. 2015) in influencing their views and maintaining their support.

Loyalty of the administrative agent to the political principal is a key norm across parliamentary systems (Hustedt and Salomonsen 2014; Christensen and Opstrup 2018). That loyalty is, however, to be directed towards the office the incumbent politician holds, not to the person. Although on a day‐to‐day basis these relationships are often intensive and inescapably interpersonal, their essence remains institutional. The institutional essence is that in return for their loyalty, public servants operate under the protective umbrella of the ministerial responsibility doctrine.

Astuteness is much less firmly institutionally rooted, and potentially more controversial. It has been defined as ‘deploying political skills in situations involving diverse and sometimes competing interests and stakeholders, in order to create sufficient alignment of interests and/or consent in order to achieve outcomes’ (Hartley et al. 2015). Hartley et al. (2015, p. 207) argue that senior public servants require political—but not partisan—skills in negotiating coalitions that form an authorizing environment for the department and its policies. In doing so, top administrators must carefully manage the ‘zone’ between themselves and the minister.

## 3 STUDYING ADMINISTRATIVE WORK: DESIGN AND METHODS

To answer our research question, we present three case narratives exemplifying elite administrative work in the Dutch core executive. All are set in the Dutch prime minister's office (in Dutch: *Kabinet Minister‐President*). This is a small but pivotal office consisting of the prime minister, one ministerial adviser (partisan), a permanent secretary (nonpartisan), and 15–20 nonpartisan senior advisers known as ‘councillors’.

Our contribution is to highlight the felt realities of managing up when the stakes are high and there are no easy answers. This narrative approach is unique in the literature, which seldom sees ‘public servant stories’ published (for a review of narratives in public administration see Borins 2011). The narratives are retrospective interpretations of sequential events, from the viewpoint of a protagonist (Ospina and Dodge 2005, p. 145; Van Maanen 2011). The aim is not to generalize, or to provide a benchmark for other public servants, but to show the complexity and interdependency of the job as it unfolds in a ‘real life’ context permeated by political turbulence.

The narratives are drawn from 31 hours of interviews with a single top public servant with 27 years in chief executive roles, including a long stint at the helm of the prime minister's department. This experienced and authoritative civil servant served multiple prime ministers. The interviews focused on the craft of senior public service leadership, seen through the prism of how the interviewee had experienced and acted in three ‘hard cases’ at the interface of politics and administration throughout his career. As interviewers we sought to inductively draw out the senior public servant's beliefs, dilemmas and practices (Bevir and Rhodes 2006) through semi‐structured conversations and ‘ethnographic’ sensitivity (Boswell et al. 2018, p. 60).

For corroboration purposes as well as to get alternative perspectives on the same sequence of events, we conducted eight interviews with our main informant's key contemporaries. Interviewees included a (former) prime minister, a cabinet minister, another political office‐holder, a political assistant to the prime minister and four senior public servants. All interviews were conducted by the authors between 2013 and 2017. They typically took between 75 and 120 minutes and were digitally recorded and transcribed verbatim. All qualitative data were subjected to thematic analysis (Braun and Clarke 2006) in a collaborative effort by both authors. The excerpts cited below have been translated to English by the authors. Where appropriate we complement interview data with reference to other sources (e.g., government documents or media reports).

### 3.1 Three tales of managing up

Through storytelling we draw out the practical implications of doing ‘responsiveness’ and ‘astuteness’ amidst turbulence for the permanent secretary's work, and how this relates to the three rules of the political–administrative game. The first tale is about the gear shifting that needs to be performed when government turnover is taking place. The second is about ‘settling’ the relationship with a new minister—and their adviser. The third concerns the management of blame.

#### 3.1.1 Absorbing political change

In 1994, a new Dutch government took office. Three parties—Social‐Democrats, Liberals and Liberal‐Democrats—formed a cabinet without any Christian‐Democratic parties, a novel situation in Dutch politics. It would be the start of eight years of pacified governance, corroborated by electoral success in 1998. There was little animosity among the coalition parties. The erstwhile prime minister reflects on the 1998 elections:

Oddly—due to lack of alternatives—there was unanimity about the continuation of the cabinet after the elections. Before the elections, there wasn't any debate either, whether that should include the Liberal‐Democrats. Yes, during the final televised debate [Social Democrat) I even said that it would be too ungracious to punish the Liberal‐Democrats too hard, and that they deserved a vote too. It's exemplary of the bygone political climate. (Interview September 2015)

Its policy paradigm resembled much of what was going on elsewhere in the world. The Dutch government policy closely aligned to Clinton's in the USA and Blair's in the UK, coined as the ‘Third Way’. This was the zenith of New Public Management philosophy where government would ‘steer’, not ‘row’, resulting in privatization and market incentives in the public sector (Pollitt and Bouckaert 2017). With the PM's blessing, the Board of Secretaries started a project called ‘Strategic Explorations’. They studied major public policy themes that had dominated the coalition's agenda during the past two terms: healthcare, social policy, education, knowledge economy, and tax reform. ‘Our’ secretary was a key advocate of the hands‐on approach taken by the secretaries in performing this rather exceptional joint exercise:

I still consider it a proven method for realizing breakthroughs: Five permanent secretaries in a room and no assistants … I'm not a terribly good writer, but I like to write a few paragraphs every now and then. It gives you a chance to really articulate your own thoughts in a strategic document such as the one we were producing.

Although they had had hopes of political impact with their strategic reconnaissance, the Secretaries took pains to avoid making it seem an ‘ideological’ exercise. They included multiple scenarios and formulated arrays of options, eschewing a single dominant frame. That said, the timing of the operation coincided with the political cycle of the incumbent coalition government and its veteran PM, who at the time of the initiation of the review were riding high in the polls.

Publishing such documents allows for them to be used in electoral programmes and cabinet agreements. They help focus public attention on key issues and underpin societal debates with robust analysis, instead of fleeting issue management and political campaign rhetoric. I was convinced that initiating the Strategic Explorations was an attempt on this PM's part to create a substantial policy agenda for a new cabinet, possibly his own.

It was not to be. In his interview, the former prime minister reflected on on the ‘strategic explorations’:

We wanted to shed light on a number of structural long‐term developments which the country faced, and the respective policy answers to them. But, in the fall of 2001 and the early months of 2002, the momentum for such an approach simply evaporated. People started to anticipate the collapse of the coalition instead.

In August 2001, less than a year before the next general election, the prime minister had announced that he would step down as party leader ahead of the next election and would not seek further public office. Two weeks later, the 9/11 attacks in New York City rocked the world and brought identity politics (issues such as non‐Western immigration) to the fore. In March 2002, the political upstart, the charismatic populist Pim Fortuyn, whose comments about Islam had skirted around the edges of what law and custom would allow, caused a major upset in the municipal elections in Rotterdam, obliterating in one fell swoop the age‐old Social‐Democratic hold on power there. A month later, a few months before its term of office would have ended, the government resigned after a damning report on the genesis and management of the Dutch peacekeeping operation in Bosnia that ended in the genocidal killings at Srebrenica in the summer of 1995. Another month later, days before the general elections Pim Fortuyn, whose party (*Lijst Pim Fortuyn*) was heading for a spectacular election victory and who might well have become the new prime minister on the strength of it, was assassinated by a radicalized left‐wing activist.

Dutch politics was in turmoil. Between October 2001 and March 2002, the coalition's standing in the polls plummeted, and the events of April and May 2002 instilled a sense of crisis, even chaos. Hailed for years as a steady, competent, reforming government, the ruling coalition's reputation was all of a sudden in tatters. It was a shock that reverberated right at the centre of power. The permanent secretary observes:

I don't think anyone could have predicted how rapidly the drama would unfold: the PM's status dropped from 75 per cent confidence to a maligned figure of the past in just a few months. That was a major turn. It was unprecedented in Dutch politics. We talked about it at PMC and in the Board of Secretaries. We frantically tried to come to terms with what was happening. I suppose it was no different in the run‐up to the 2017 general election, after Trump's electoral success in the USA. In such a situation, I simply start to prepare for anything that can happen—I began to envisage a situation in which the current PM would be no longer prime minister, and in which his successor might well be a very different kind of politician with a drastically different programme and governing style.

The prime minister had become a lame duck almost overnight, facing death at the polls (Shaffir and Kleinknecht 2005). His political capital had shrunk. The PM reflects on the schism between the cabinet's policy and the ubiquitous electoral promises and sentiments about upcoming policies:

I think it was very difficult for public servants to fill this gap. That even these ‘Strategic Explorations’ would collapse, says it all. The universal, implicit, attitude was that after the 2002 elections, a new government would start with a completely clean slate, most likely one that repudiated what had preceded it. The incumbent government no longer had any influence on the future agenda, despite the public servants’ best efforts with their ‘Explorations’ exercise. (Interview Prime Minister, September 2015)

In the spring of 2002 his coalition's political implosion combined with the moral burden of the Srebrenica drama took a heavy toll on the prime minister. At a low point, he called on his permanent secretary, who recalls:

For him, the responsibility was personal … We talked for one and a half hours, at the prime minister's office. He said: ‘I have a dilemma. My wife wants me home, but the party wants me to stay on.’ … He was very specific. He did not ask for political advice, but for professional advice. Such a question, such a conversation was one‐of‐a‐kind in my career. Shortly afterwards, it was back to normal—distanced and professional.

In this narrative of prime ministerial decline and anticipation of new undefined political regimes, we see a permanent secretary at work in the prime minister's office. The relationship between the PM and the secretary suddenly became unusually close. Normally strictly business‐like and professional, the PM became personal and emotional. In that political and personal predicament, the PM sought out his permanent secretary, a public servant, rather than his partisans, his designated successor or the deputy prime ministers, to contemplate the possibility of resignation.

The dichotomy doctrine offers no script for the senior public servant called upon to act in these circumstances. What do ‘responsiveness’ and ‘astuteness’ mean when the political edifice of the government one serves is crumbling? Rather than maintaining the façade of the dichotomy, the PM and the permanent secretary act in the spirit of the unwritten rules of Dutch political–administrative interaction. Amidst the turbulence of the era, the PM and the secretary demonstrate mutual respect for the roles each has to perform. As the permanent secretary and his colleagues sought to provide stewardship of the long‐term policy agenda and the PM gave them ample discretionary space to do so by framing the ‘Explorations’ as an administrative project rather than a political one. In turn, the permanent secretaries were careful—although ultimately unsuccessful when it was met with frosty indifference from the newly emerging political masters—to avoid it becoming unduly politicized.

Meltsner (1988) argues that advisory relationships can be understood in terms of four stages: entry, consolidation, harvesting of results, and pre‐exit. The pre‐exit stage requires a delicate balancing act, and we see this at work in the story. The permanent secretary is loyal and dedicated, but inevitably bound by the professional nature of the relationship. When it becomes clear that the incumbent government is on its way out, the secretary has to gear up the department for serving its successor. Political astuteness in these circumstances demands operating on parallel tracks: staying the course with the incumbent, but building bridges towards an as yet indeterminate future without that incumbent and in possibly—in this instance dramatically so—a radically altered political constellation.

Even experienced senior public servants can find it hard to switch gear, as the secretaries did when they were taken aback by the vacuum in which their cherished Strategic Exploration exercise landed in the political turbulence of spring and summer of 2002. ‘Serially monogamous’ professionals they may aspire to be, but coming out of a collaboration with a long‐serving government even senior public servants may develop affinities with the actors, policy paradigms and governing styles they have worked with for so long and that they have not only become used to but have somewhat invested in. They need to overcome their inertia to be flexible enough to adapt responsively to new politicians and their worldviews, priorities, words, capacities, styles and temperaments and establish yet another ‘public service bargain’ with them (Hood and Lodge 2006).

#### 3.1.2 Building new relationships

The turbulent general election of 2002 proved to be an unprecedented electoral landslide. The Christian‐Democratic party won the largest number of seats, rapidly forming a coalition with the late Pim Fortuyn's party, and the Liberal Party. Enter a backbencher without prior ministerial experience as prime minister. For public servants, ministers come and go, but change is rarely easy. Building an effective working relationship is key. Public servants try to ‘read’ the minister (see also Noordegraaf 2000). How does this minister work? Does s/he read papers or prefer to talk it through? Does s/he prefer structured deliberation or free brainstorming? What is his/her standing in the party, cabinet or media? What is his/her work ethos, integrity, any apparent fallacies? How can we realize policy goals with this minister? This professional working relationship is not a given, so triadic relationships must be built: minister, ministerial adviser, and permanent secretary. In 2002, there was yet little tradition of ministerial advisers in the Netherlands (Van den Berg 2018). From 1994, a number of ministers had ‘parliamentary assistants’ who liaised between the cabinet members and their respective parliamentary party, but they were strangers within the department. The secretary recounts the challenges of the start‐up phase:

The puzzle was: how do you support someone in such a crucial office who has no prior executive, ministerial or otherwise, experience, and who is simultaneously expected to show leadership? How do you get him in position? These first months were: being there 24/7, analysis, making lists, identifying key messages, being present, helping, steering.

After a rocky start, more exploration needed to be done to establish how the department could best meet his needs, and how the minister could carve out a leadership style that befitted him:

We often talked about leadership. What makes someone a leader? I sensed that all media analyses would focus on his leadership, asking: what sort of a leader is our new prime minister? So, we discussed: what are your strengths? How does that fit your leadership? What do we need? We got it down to ‘connective and servant leadership’. He said: ‘write that down, I'll use it in Parliament’. He was the leader, and he had to grow into that role. He worked very hard—and succeeded.

And:

You must learn to read him—how to get your message across to a new minister. We managed, because the prime minister was very open and approachable.

In this instance, the differences between the former and the current minister were quite considerable. An experienced councillor compares and contrasts:

The former minister was not a fan of modern technology. Along with the new minister came BlackBerrys. He was focused on image, media performance, ‘door stops’ and ‘oneliners’. It dawned on us that this was an entirely different person. His predecessor read everything and did not mind expansive briefs. The latter wanted everything summarized. He had less time—but he was an avid mobile phone user. Suddenly, we no longer knew who had spoken to the PM. (Interview October 2015)

Earning standing with a new minister proved to be a struggle. Having the ear of the PM was not solely the permanent secretary's privilege. The new PM's ministerial adviser injected himself into the departmental court, shaking its equilibrium (Rhodes 2017; see also Hustedt et al. 2017):

A new development was the political adviser who came along with the prime minister. Our permanent secretary heavily objected to that. He was a traditionalist. He champions the ‘neutral public service’ narrative. He didn't want politics and administration to mix. … He was very clear: our advice is professional, not political. (Interview Councillor, December 2015)

The strongly partisan and ‘PR’ focus of the new generation of ministerial adviser came as somewhat of a shock to the system:

In the media, policy successes were sold as victories at the expense of coalition partners. ‘Spinning’ had reached the Netherlands. In the next cabinet [2007–10] Social‐Democrats used a similar strategy. There was much spinning, to the detriment of cabinet unity and policy coherence. The first political assistant [2002–07] still had a grip on that game, he was aware of the consequences. His successors were not. By that time, the Government Press Office had lost control. (Interview Director‐General Government Press Office, September 2015)

The PM had multiple sources of advice: the partisan (here embodied by the ministerial adviser), the public service (the permanent secretary and councillors), his personal network (e.g., a befriended professor and senator with expertise in constitutional matters). Realizing his propensity to consult the latter, the department adapted and began to include his advice beforehand. The secretary: ‘when we learned that on all constitutional matters the PM sought out this professor, our councillor started to call him beforehand—we called it “informal coordination on behalf of the prime minister”’. And yet the department wanted to remain the pivotal adviser to the PM as it believed that this served him best. The secretary cast a weary eye on the PM's proclivity to consult widely from his political and social networks: ‘I often said to the prime minister: “trust your own department”’. The PM's ministerial adviser had his own take on the dynamics:

Obviously, he [permanent secretary] was both literally and metaphorically bigger than this freshman prime minister, but then, all of a sudden, the prime minister was roaming the halls of the office and meeting with ‘his’ people, bypassing the permanent secretary. However, it fitted the modern times, and the councillors loved it. (Interview September 2015)

He goes on to say:

I wondered whether the permanent secretary of the PMC appreciated that, because the prime minister had made a decision and he had not been the chief adviser [in a specific dossier]. I think he was quite sceptical—it confirmed his view that the PM was inexperienced. (Interview September 2015)

The start of this prime minister's tenure followed what is considered the ‘normal’ script in Anglo‐Saxon systems: politics (the prime minister and his political entourage) are on top; the public service is on tap. In the Dutch system, this had not been so, and therefore the secretary was sceptical about what he perceived to be a break with tradition. The permanent secretary has an innate respect for the prime minister and his office, but clearly saw the assertiveness of the ministerial adviser—who had been the PM's campaign leader in the election campaign that had swept him into office—and the symbiotic relationship he had forged with the PM as an anomaly. And yet, as ‘responsive’ public servants he and his staff had little choice but to adapt: to learn to ‘read’ the new minister and to learn how they could work most effectively for and with him. In turn, the PM seemed to initially prefer his political entourage over the department. Over time, new rules of engagement were negotiated between the actors in the triad (Van den Berg 2018).

Responsiveness here meant that the permanent secretary—possibly against his professional judgement—had to accept the presence of an unusually active and powerful ministerial adviser in the departmental court, simply because the PM wanted him there and authorized him to play that role. Astuteness then means being proactive in adapting to the new PM. The story of how the PMC adapted by incorporating the external professor's advice on constitutional matters in their own advice to the PM shows how the department regained a grip of the advisory system while being attentive to the PM's preference of hearing this professor's view on the matter. Astuteness in this instance also entailed ‘training’ the PM. The permanent secretary felt responsible for the coordination of cabinet policy and tried to equip the new PM to perform that role effectively, for example, through instigating reflective conversations on leadership.

#### 3.1.3 In the eye of a media storm

On Saturday 24 May 2004, the prime minister's residence caught fire. The seventeenth‐century building was undergoing refurbishment at the time. A painter and a resident staff member escaped in time, but a second painter got trapped in the fire and died.

Formally, political responsibility for the conduct of the government's facility management service, which was the legal owner of the building and in charge of the refurbishment, rested with the minister of public housing. However, the public eye was firmly trained on the role of the resident of the building, the prime minister—and his department. A series of investigations ensued, seeking to determine what ignited the fire and what caused the fatality. The secretary received a brief internal memo on 27 June 2004 suggesting that experts disagreed about the cause of the fire, but that tentative test results suggested that the painters might have used prohibited highly flammable liquids (‘thinner’) which along with antique gobelins in the room had caused the fire to escalate so quickly. The secretary PMC decided not to inform the prime minister and the public prosecutor at this stage. He took a formal view of the lines of accountability that ought to be at play:

The housing ministry was responsible, not the prime minister's department. Ministerial responsibility rested with them, not us. Also, these were tentative, disputed research findings, and further research was being conducted. And emails from the housing ministry that I had received suggested that no rules had been violated by government officials.

In 2006 a judge ruled that the contracting painters company had indeed used unauthorized thinner and that there was no evidence to suggest that government officials had prior knowledge of this use (The Hague Court 2006).

However, opposition members of parliament and a number of journalists kept the case in their sight and continued digging. In response to ongoing media reports and political clamour the prime minister asked the public prosecutions office to start a new inquiry. Months later, in November 2008, the secretary, who had moved to head up a different department in 2007, was called in for a hearing. At this time, he received word that, in addition to the aforementioned memo, there had been a second memo substantiating the research findings and the crucial role of the wall drapes in speeding up the fire. These findings had been corroborated by foreign researchers. The secretary reflected:

My feeling was that if I had been given that full memo back in 2004, I would have chosen to communicate these research findings—no matter how tentative and disputed—to the PM and the public prosecutor.

But that hadn't happened, and in the meantime the story would not die. In June 2009, an internal non‐paper from the department was leaked to journalists. The non‐paper included the phrase that the then secretary had been ‘given notice’ of the existence of a second memo written a few days after the first—implying that he had known more about the forensics of the incident than he had conveyed to the PM. The secretary emphatically denied that this was the case:

Registration of the flow of communications was routinely done very carefully at the PMC. We called it the ‘Rob Visser doctrine’, named after a very constitutionally prudent councillor: always keep track of and record who has read which papers when. Administrative behaviour must be traceable at all times. That's not just professional good practice; it is essential to buffer ministerial responsibility.

This narrative is one of a cold case becoming a media storm and a political bone of contention. One television news programme suggested that there had been a ‘cover‐up’ by departmental staff while simultaneously portraying the name and a picture footage of the then secretary.^1^ Parliament was up in arms and called the prime minister to account for what had happened.

The doctrine of ministerial responsibility prescribes that the minister is responsible for any and all matters in their portfolio (for interpretations of the doctrine in Westminster systems see Woodhouse 1994; Rhodes et al. 2009). Although formally this would mean that the housing minister and not the PM should have conducted the debate, the political realities at the time were different. The question now became what the PM would do. In keeping with the rules of the game of the politics–administration interface in the Netherlands, would he step up, defend his department's actions and absorb any blame directed at it by taking responsibility? Or would he throw the public servants—conspicuously including his erstwhile secretary—to the wolves in an effort to deflect blame? A turbulent debate ensued on 23 June 2009. A significant proportion of parliament expected acknowledgement of error and some form of contrition, while the secretary expected his minister to take the heat. During the run‐up to the debate, the secretary felt caught in the crosshairs. He reflects on his internal and professional struggle:

I could not defend myself. As a public servant you have one hand tied behind your back. I knew this—but now I felt how frustrating this is. I could have chosen to break protocol and argue my case in public, or leak. Given the heated atmosphere, I would have been on prime‐time television. But that would undermine the [doctrine of] ministerial responsibility. It would be unthinkable to deny my public service loyalty.

A fellow permanent secretary explained that when these things happen, the very core of one's identity is being questioned:

It's a big deal when it is implicitly or explicitly implied that you are (in part) answerable for someone's death. My assumption is that this is not about professionalism, but rather about humanity and integrity. It is not a rational debate, yet feelings are facts. What is the craft without passion? Without values? I would think that this is what preoccupied him the most. (Interview September 2015)

The PM tried to steer a middle course. In one breath he defended the integrity of the public service and that of his own department in particular (rejecting the notion that there had been a cover‐up). In another breath he criticized the professionalism it had displayed in this instance, using harsh terminology—‘imprudent, incorrect, and insufficient’—in doing so. He succeeded in placating the opposition, but did nothing to counter the media and political assault on the reputation of the public service.

The secretary has been dragged into the public spotlight and into the heart of political controversy. Rather than a steady hand in the background helping the minister through parliament, he now has to be helped by the minister to calm the whipped up furor of the parliament. How does one balance responsiveness and astuteness when the going gets tough? The resolution for the struggle is not self‐evident. For public servants, their currency is their professional reputation and status among their peers. This is what's at stake in the story—for the secretary the case boils down to the PM's political leadership and role‐taking: does he prioritize hoarding his political capital or does he stick to the public service bargain?

There is a larger issue here, however. Early on, the secretary had made a conscious decision not to inform the PM about the contents of a memo based on his judgement that its status was premature. This decision became a pivotal issue in the ensuing political debate. It was his job to monitor and manage which information reached the PM. Access to the PM is carefully managed by his office. Determining what is, and what is not, is the prerogative of the secretary. The rules of the game of political–administrative interaction suggest that the PM should trust the secretary and allow discretional space to make such decisions.

In this case, the public servant's discretionary judgement was questioned by public and political scrutiny, and this was exacerbated by the public framing of the issue as one of integrity (the ‘cover‐up’ charge). The secretary could have opted to wage his own public defence, by making a press statement or giving an interview. Had he done so, the secretary argued, then he would have pre‐empted ministerial responsibility from being enacted in the right forum (parliament) at the right time (the designated debating slot) by the right actor (the prime minister). Moreover, Dutch parliamentarians generally have little appreciation for that manifestation of political astuteness on the part of a public servant. So the secretary chose to sit still and await his fate. This adherence to the classic rules of the game is interesting in light of recent scholarship about senior public servants’ public leadership. Grube (2014, 2019) argues that top public servants in Anglo‐Saxon democracies use ‘voice’ and not just ‘loyalty’ or ‘exit’ when dealing with issues of major concern to them. In this instance, our secretary considered speaking up publicly to be in violation of his loyalty to the office of the PM.

## 4 NAVIGATING THE DICHOTOMY: WHAT HAVE WE LEARNED?

The central puzzle in this article was how top‐level public servants navigate the political–administrative dichotomy. We asked how they ‘manage up’, and ‘administrative responsiveness’ and ‘political astuteness’ take shape in day‐to‐day administrative work and behaviour. Starting from the dichotomy doctrine and its critiques, we have discussed three ‘rules of the game’ in Dutch political–administrative interaction: mutual respect, discretionary space, and reciprocal loyalty. Our case stories illustrate the everyday complexity of this institutional relationship, before we discussed implications for the craft of top‐level public servants. The first case narrative illustrates how the sudden imminence of an incumbent government's political decline punctuated by a sequence of crises deepened the professional bonds between the PM and the secretary, whereas the second narrative demonstrates how challenging the establishment of such bonds at the outset of a new government's life can be. Responsiveness and astuteness take shape over time. As change can be sudden, institutional inertia must be overcome to adapt to new political Zeitgeists and the new types of actors and rules of engagement that accompany them. Public servant behaviour is related to events and incidents as well as political and policy cycles.

Second, where the secretary had been socialized during an era when political–administrative relations were largely dyadic, they are no longer so. The introduction of ministerial advisers—who arrived on the Dutch scene relatively late, and in modest numbers compared to most other parliamentary democracies (Van den Berg 2018)—has complicated the picture. Particularly when ministers have more of them, accord more weight to their advice, or allow them more expansive views of their roles, core executive relationships change. The public servant–minister dyad effectively becomes a triad (Shaw and Eichbaum 2016), if not a much broader polycentric network that includes consultants, lobbyists and media. The craft of top‐level public servants has come to include ensuring that public service advice continues to occupy a prominent role within these more complex configurations of consultation and influence.

Third, media and political framing of incidents can put the public service suddenly and squarely into the spotlight of ministerial accountability, taxing the traditional rules of engagement between political office‐holders and public servants. Perhaps it is no surprise, therefore, that students of executive politics, political accountability, ministerial careers and public service leadership have started to look into these dynamics of blame management much more intensively (Hood 2010; Brändström 2016; Hinterleitner and Sager 2018; De Ruijter 2019). What they have learned is that more wedges can be driven into the traditional compact between politics and public service, challenging public servants’ ongoing balancing act of combining responsiveness with astuteness. As the third case narrative suggests, this opens up a new dilemma: when their past behaviours, including their advice‐giving to ministers, are being scrutinized in the context of blame games, and when ministers are not inclined to take responsibility for the public service's actions, what are senior public servants to do? Sit still and await their fates (loyalty), offer themselves up as a scapegoat and resign (exit), or publicly defend themselves (voice)?

Our account highlights the intimate and backstage practices of government elites and suggests that the top public servant's craft involves constant calibration and balancing of responsiveness and astuteness. Our contributions to the literature are twofold. First we complement the institutional, biographical, and (quantitative) survey literatures with rare original qualitative (ethnographic) evidence. We tell ‘public servant stories’ which help us understand the world of high politics (and indeed high administration). Our second contribution is that we illustrate the practices of this particular public servant as a ‘craft’. The practices are not just (bounded) rational behaviour, but crafted practices that make sense in the particular ‘local’ context and probably much less elsewhere. It is this craft that enables public servants to make sense of the situation and to act when there are no easy answers.

The detailed approach taken in this article allows the reader a taste of the felt realities of high stakes, political death and survival, relentless accountability, and parliamentary scrutiny which (in part) instigate the call for political advice, entourages, and blame deflecting to deflect pressure. It is in this world that top‐level public servants need to (re)discover and negotiate their role and added public value.

Further research on the political–administrative dichotomy and the work of public servants should take changing mores into account and examine their implications for public service professionalism and political–administrative relations. It should focus on the challenges, dilemmas, and coping patterns of public servants through detailed studies of their efforts to balance ‘responsiveness’ and ‘astuteness’ in an increasingly turbulent, incident‐prone and unforgiving political environment.

## Footnotes

1 Dutch Media file online on: http://media.rtl.nl/media/actueel/rtlnieuws/2009/catshuisbrand-coverup.pdf
